# An Integrated Approach Using Temperature–Humidity Index, Productivity, and Welfare Indicators for Herd-Level Heat Stress Assessment in Dairy Cows

**DOI:** 10.3390/ani15223341

**Published:** 2025-11-19

**Authors:** Roman Mylostyvyi, Olena Izhboldina

**Affiliations:** 1 Department of Animal Feeding and Breeding Technologies, Dnipro State Agrarian and Economic University, S. Efremov Str. 25, 49600 Dnipro, Ukraine; 2 Department of Technologies of Production and Processing of Livestock Products, Dnipro State Agrarian and Economic University, S. Efremov Str. 25, 49600 Dnipro, Ukraine

**Keywords:** dairy cows, temperature–humidity index (THI), natural ventilation, heat stress, milk productivity, clinical indicators, analytical algorithm

## Abstract

The temperature–humidity index (THI) remains one of the most widely used tools for assessing heat stress in dairy farming; however, its application is often limited by methodological inconsistencies and insufficient integration with welfare indicators. This study proposes a unified analytical framework for evaluating thermal load at the herd level by combining daily THI values with productivity, feed intake, and clinical indicators such as mastitis and lameness. The analysis was based on two years of herd-level data from a commercial dairy farm with naturally ventilated barns. General linear models (GLM) were applied to assess both direct and delayed effects of heat stress and to compare model reproducibility across years. The results confirmed that maximum daily THI had the strongest association with milk composition and dry matter intake, while cumulative heat load and elevated night-time THI contributed to increased mastitis and lameness incidence. The inclusion of welfare indicators substantially improved the explanatory power of THI-based models, providing a more biologically relevant assessment of heat stress. The proposed framework enhances the accuracy of herd-level monitoring and supports the development of predictive models for welfare-oriented management in dairy systems.

## Simple Summary

Dairy cows are highly sensitive to heat stress, particularly in naturally ventilated barns where indoor conditions depend on the external climate. While the temperature–humidity index (THI) is widely used to estimate thermal load, traditional approaches often rely on average or maximum values alone, overlooking daily patterns and delayed physiological responses. This study presents a five-step analytical approach that combines THI data with herd-level records on milk production, feed intake, mastitis, and lameness to evaluate the overall impact of heat stress. By integrating environmental and clinical indicators, the method improves the detection of heat-related risks and provides a more accurate assessment of cumulative effects on productivity and welfare. This approach supports practical decision-making in dairy systems and may serve as a foundation for predictive models and real-time monitoring strategies.

## 1. Introduction

Heat stress (HS) is recognised as one of the key limiting factors affecting the productivity and welfare of dairy cows, particularly under conditions of increasing climatic variability. Excessive thermal load disrupts thermoregulatory balance, reduces feed intake, and consequently lowers milk yield. It may also compromise milk quality by altering its fat and protein content [1,2]. Beyond its direct effect on productivity, HS increases the risk of metabolic disorders and clinical conditions, especially mastitis and lameness [3,4].

The temperature–humidity index (THI) is the most widely used integrated indicator for the quantitative assessment of HS in dairy farming [5,6]. However, the selection of input parameters for THI calculation remains subject to methodological debate. In practice, both average and maximum values of temperature and humidity are applied [7]. Average values reflect the overall thermal load over a given period, whereas maximum values capture stress peaks that may be particularly critical for high-producing animals. The combined use of both metrics enables a more comprehensive evaluation of microclimatic effects on the herd [8].

An important aspect of heat load assessment is the daily variation in THI and the effectiveness of night-time cooling, as an insufficient nocturnal temperature drop limits physiological recovery and leads to cumulative thermal stress over consecutive days [9]. This factor is particularly relevant in naturally ventilated systems, where microclimate regulation is restricted, and night cooling often remains the only passive mechanism for mitigating HS.

Sensitivity to HS not only manifests in immediate reductions in milk yield and quality but may also be mediated by indirect and delayed physiological pathways. These include reductions in dry matter intake (DMI), increased incidence of clinical conditions such as mastitis and lameness, and cumulative stress responses over time. Notably, such effects may persist or intensify even after thermal conditions have returned to normal, suggesting the presence of lagged impacts of THI on productivity and welfare [10,11]. This underscores the importance of integrating both direct and indirect indicators into analytical models, including time-lagged predictors and clinical health variables, to better capture the complex dynamics of heat stress at the herd level.

Seasonality remains an important background factor influencing the variation in productivity and welfare; however, it does not always align with periods of elevated thermal load. The integration of THI into multifactorial analysis enables a quantitative distinction between the effects of calendar seasons and actual microclimatic stress [12].

We hypothesised that the impact of heat stress on milk yield and composition is mediated not only by direct thermal effects but also by indirect pathways, including reduced dry matter intake and increased incidence of clinical conditions such as mastitis and lameness. Furthermore, these effects may be delayed, manifesting even after the immediate heat load has subsided. Therefore, incorporating both thermal indices and welfare-related variables into regression models may improve the accuracy and biological relevance of such assessments.

The aim of this study is to present a five-tier methodological framework that integrates THI parameters and welfare indicators into a multifactorial analysis of heat stress effects on dairy herd productivity. This approach was applied to two years of herd-level data from a commercial farm with naturally ventilated barns, enabling the evaluation of lagged effects, cross-year reproducibility, and the practical implications of prolonged heatwaves for herd health and performance.

## 2. Materials and Methods

### 2.1. Study Site, Housing Conditions, and Meteorological Data Sources

The study was conducted at the Ukrainian Dairy Company LLC (50.4912° N, 31.4923° E), one of the largest dairy farms in Ukraine, located in the Kyiv region. The climate in this area is classified as Dfb (humid continental climate with warm summers) according to the Köppen system, with an average temperature of +20 °C in July and −5 °C in January. The farm is a registered breeding enterprise for Holstein cattle and holds the official status of a designated raw material zone for the production of infant nutrition. This status was granted by the Ministry of Agrarian Policy and Food of Ukraine following a state-level examination and confirmed by the State Service of Ukraine on Food Safety and Consumer Protection.

The milking herd comprises approximately 4000 cows, with an average annual milk yield exceeding 10,000 litres per cow. The cows were housed in naturally ventilated barns oriented along the northeast–southwest axis. Each barn comprised four pens accommodating production groups of approximately 140–150 cows, with dedicated feeding alleys and automatic group drinkers. Resting areas were equipped with two rows of cubicles with sand bedding (1.2 × 2.5 m), providing comfortable lying surfaces for the animals.

The barn structures featured a dual-pitch roof with a ridge height of 9 m, sandwich panel insulation, and 3.0 m high side walls with reinforced concrete bases and retractable canvas curtains to facilitate natural ventilation. Additional climate control was provided by a supplemental axial fan system, automatically activated when indoor temperatures exceeded +19 °C. During hot periods, the fans operated continuously. Airflow velocity in the cubicle zones ranged from 1.5 to 3.6 m/s and was periodically monitored using a hot-wire thermoanemometer GM8903 (Benetech, Shenzhen, China).

The total mixed ration (TMR) is offered year-round and consists of corn silage, grass silage, hay, straw, rolled grains, oilseed meals, dried beet pulp, and mineral-vitamin supplements. Based on monthly feed analyses, the average chemical composition of the ration is as follows: dry matter 40–48%, crude protein 16–18%, neutral detergent fibre (NDF) 30–35%, and metabolizable energy (ME) 10.3–11.0 MJ/kg DM. The ration is adjusted monthly to meet the nutritional requirements of high-yielding Holstein cows in accordance with NRC (2001) guidelines [13].

Cows are milked in automated DeLaval Cascade milking parlours with 72 stalls, and computerised identification and monitoring systems (DairyComp 305, Valley Agricultural Software, Tulare, CA, USA; Alpro, DeLaval, Tumba, Sweden). Each cow is fitted with an activity sensor that enables health monitoring. Herd-level observations were carried out over a two-year period. Data from 2023 were used to develop the methodological framework, while 2024 data served to test hypotheses and validate the proposed methodological approach.

All data collection procedures were conducted without compromising animal welfare and did not involve any handling of live animals, as the study was based on the analysis of production and statistical records, in accordance with the principles of bioethics and good production practice.

Meteorological data (air temperature and relative humidity) required for the calculation of the temperature humidity index (THI) were obtained from the Boryspil International Airport weather station (UKBB), located approximately 20 km from the study site. The data were provided by the Ukrainian Hydrometeorological Center (UkrHMC) and are publicly available on its official website. All retrieved records underwent standard quality control by UkrHMC and were used without additional correction. Measurements were recorded at fixed hours: 00:00, 03:00, 06:00, 09:00, 12:00, 15:00, 18:00 and 21:00, with a 3 h interval (eight records per day), resulting in 2920 paired temperature and humidity records for 2023 and 2928 records for 2024. Considering the importance of nocturnal recovery in cattle, a biologically justified approach is to distinguish thermal load into nighttime (21:00 to 06:00) and daytime (06:00 to 21:00) periods, with average THI values calculated separately for each. This approach helps to assess whether animals experience sufficient cooling during the dark hours when thermal stress is expected to decline.

### 2.2. Data Acquisition, Preprocessing, and Coding

Production data on milk yield, dry matter intake (DMI), and feed efficiency (FCR) were obtained from the herd management software DairyComp 305 (Valley Agricultural Software, USA) and processed at the herd level as daily averages. Dry matter intake (DMI, kg/cow/day) was calculated based on daily feed delivery and refusal records. Feed was distributed using a Peecon Biga Mammoet feed mixer wagon (Peecon, Etten-Leur, The Netherlands) with an integrated electronic weighing system. Orts were collected and weighed after feeding, and net intake was calculated as the difference between the total feed offered and refusals. The DMI per cow was then calculated by dividing the net intake by the number of lactating cows in the herd. All records were logged in the herd management system, with calculations overseen by the farm nutritionist.

Welfare indicators (mastitis, lameness) were available at the herd level and analysed accordingly. DairyComp 305 automatically flagged cows with decreased milk yield or abnormal performance parameters, prompting clinical examination by veterinary staff. Mastitis diagnosis was confirmed via inspection, palpation, and the California Mastitis Test (CMT). Lameness was identified based on gait abnormalities, posture, and reluctance to bear weight. The incidence of mastitis and the prevalence of lameness were calculated monthly as proportions of the total lactating herd. Although assessments were conducted daily as part of routine health monitoring, data were summarised monthly or upon the detection of clinical signs.

The THI was calculated using the classical NRC 1971 formula, based on air temperature (T, °C) and relative humidity (RH, %) measured at 3 h intervals [14]:

THI = (1.8T + 32) − ((0.55 − 0.0055RH) × (1.8T − 26))(1)

Considering the importance of nocturnal recovery in cattle, a biologically justified approach is to distinguish thermal load into nighttime (21:00 to 06:00) and daytime (06:00 to 21:00) periods, with average THI values calculated separately for each. This approach helps to assess whether animals experience sufficient cooling during the dark hours when thermal stress is expected to decline.

In addition, from a technical perspective, the ratio between the minimum and maximum daily THI (THI min/max) offers a simplified metric that does not require hourly partitioning or processing of large data arrays. While it may not directly reflect the absolute severity of heat load, this parameter captures the amplitude of diurnal fluctuations and provides insight into the general stability or variability of environmental conditions. Although THI min/max and THI night/day are derived from the same base measurements, they were included in the comparative analysis as complementary indices to evaluate different aspects of thermal stress dynamics.

For further statistical analysis, the dataset was standardised and categorised prior to statistical processing. Continuous variables without predefined biological thresholds (e.g., milk yield, milk components, DMI, FCR) were classified into low, medium, and high levels according to the ±0.67 standard deviation (σ) rule from the mean, corresponding approximately to the lower 25%, middle 50%, and upper 25% of the distribution. Heat stress levels were defined using THI classification established in our previous study [15], where THI ≤ 67.9 was considered comfort (code 1), 68.0–71.9 mild stress (2), 72.0–79.9 moderate stress (3), and ≥80.0 severe stress (4). Additionally, the season was coded as a categorical variable: winter = 1, spring = 2, summer = 3, autumn = 4. The same classification principles were applied to the 2024 dataset to ensure consistency and enable cross-year comparison within the developed methodological framework.

### 2.3. Analytical Framework for Impact Assessment

**Figure 1:**
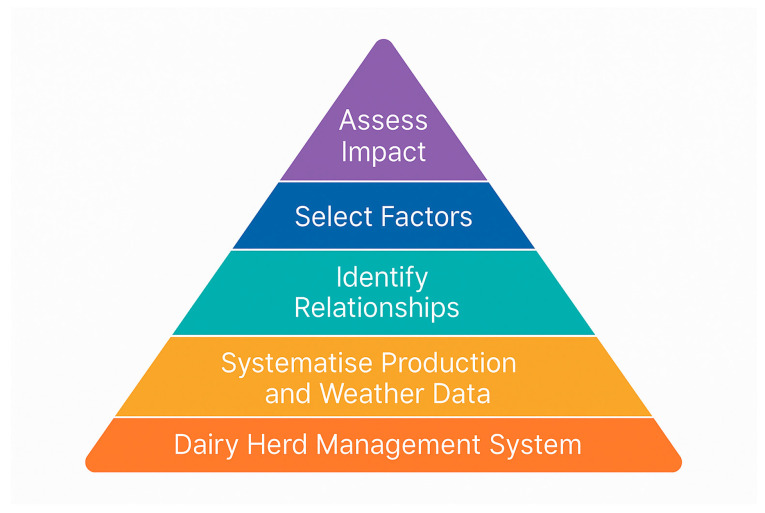
Analytical framework for assessing the impact of heat load on dairy herd productivity and welfare. The diagram reflects the sequential logic of the study design, from raw data acquisition to multivariate modelling and interpretation of both direct and indirect effects of THI.

The methodological approach to assessing the impact of heat load on herd-level productivity and welfare was developed based on a biologically grounded hypothesis: the effects of thermal stress (THI) may be mediated not only directly through productivity changes, but also indirectly through reductions in feed intake (DMI), increased incidence of mastitis and lameness, and delayed (lagged) physiological responses. To test this hypothesis systematically, a five-tier analytical pyramid was constructed to guide data processing, statistical modelling, and interpretation of results (Figure 1).

Each tier corresponds to a distinct stage of the analysis:

Dairy herd monitoring system: Ensures reliable, high-frequency collection of key performance indicators (milk yield and composition, DMI, FCR, mastitis, lameness) using automated herd-level data capture tools.

Systematisation of production and weather data: Integrates daily production traits with meteorological records (temperature, humidity, THI indices) into a unified longitudinal dataset covering two full years.

Identification of associations: Involves correlation analysis (Spearman’s ρ) between THI and performance indicators to identify relevant predictors, guide variable reduction, and explore year-specific differences and potential reversals in associations.

Factor selection: Combines statistical criteria (significance, consistency, effect size) with biological rationale (heat stress pathophysiology) to select a subset of predictors (e.g., THI max, THI night/day, DMI, mastitis, lameness) for regression modelling.

Impact estimation: Implements general linear models (GLM) with and without seasonal factors, incorporating lagged predictors to assess the explained variance (η^2^) and delayed effects of thermal stress on productivity and welfare indicators. Model accuracy was further validated using 2024 data.

This structured approach ensures that each analytical step is hypothesis-driven and contributes directly to testing the proposed mechanism of delayed and mediated THI impact. The five-tier pyramid is not arbitrary but reflects the logical decomposition of a complex multifactorial phenomenon into tractable, sequentially linked components.

### 2.4. Statistical Analysis

All statistical analyses were performed using Statistica 12.0 (StatSoft Inc., Tulsa, OK, USA). Prior to modelling, the dataset was screened for completeness and data quality. Only a negligible proportion of missing values (<1%) was identified, mostly within monthly milk composition records. These were imputed using linear interpolation, based on temporal continuity within each trait’s sequence. No imputation was performed for categorical or clinical variables.

Normality of continuous variables was assessed using the Shapiro–Wilk test. Most variables deviated significantly from normal distribution (*p* < 0.05), except for milk yield. Therefore, Spearman’s rank correlation coefficient (ρ) was used for the correlation analysis between THI parameters and performance traits. Heatmaps of correlation matrices were used to guide predictor selection for modelling.

No formal power analysis was performed, as the study was retrospective and based on two years of daily herd-level observations. Nevertheless, the dataset was considered sufficient for the applied modelling approach.

To evaluate the effects of environmental (e.g., THI parameters) and physiological (e.g., DMI, mastitis) factors on productivity, milk composition, feed intake, and welfare indicators, we constructed a series of general linear models (GLM). This method was selected instead of multifactorial ANOVA due to its flexibility in handling both continuous and categorical variables, testing lagged effects, and enabling cross-year model comparison.

Two types of model structures were used:

GLM without season (used in baseline models and for assessing pure thermal effects):

Y = b_0_ + b_1_X_1_ + b_2_X_2_ + … + b_k_X_k_ + ε (2)

GLM with season (to account for physiological and managerial shifts beyond thermal load):

Y = b_0_ + b_1_X_1_ + b_2_X_2_ + … + b_k_X_k_ + b_k+1_S_1_ + b_k+2_S_2_ + b_k+3_S_3_ + ε (3)

where Y is the dependent variable; X_1_…Xₖ are continuous or binary predictors; and S_1_…S_3_ represent seasonal dummy variables corresponding to winter, spring, and summer, with autumn used as the reference level.

Season was included as a four-level categorical factor (coded as 1 = winter, 2 = spring, 3 = summer, 4 = autumn), with autumn serving as the reference level. This structure allows for separate estimation of thermal (THI-related) and seasonal (non-thermal) effects within the same model. This model structure partially mitigates the issue of collinearity between season and THI, allowing for their simultaneous inclusion and interpretation.

Effect sizes were quantified using partial eta-squared (η^2^_x_). While confidence intervals for η^2^_x_ were not available in Statistica 12, 95% confidence intervals for parameter estimates (β) were reported in result tables (e.g., in the output tables). Model fit was assessed via R^2^, adjusted R^2^, mean absolute error (MAE), and root mean square error (RMSE). Bonferroni correction was applied to control for multiple testing.

Model assumptions (normality and homoscedasticity of residuals) were checked using diagnostic plots and residual analysis. Data transformation was not applied, except in preliminary trials where deviations were substantial.

All visualisations (seasonal dynamics, THI trends, interaction effects) were performed using GraphPad Prism 9.0 and Microsoft Excel 365. Statistical significance was accepted at *p* < 0.05, unless otherwise stated. Model structures and predictor thresholds are explained in detail in the Methods section and Appendix A.

## 3. Results

### 3.1. Monitoring of Productivity and Welfare at the Herd Level

Throughout 2023, monthly monitoring of key productivity and clinical parameters at the herd level revealed distinct seasonal patterns. Average milk yield gradually increased from 32.8 kg in January to a peak of 34.9 kg in July (+6.4%), after which, under the influence of elevated THI, it declined to 34.1 kg in October (−2.3% from the maximum) and stabilised at 33.8 kg by December. Milk fat content decreased from 4.29% in January to a minimum of 3.85% in August (−10.3%), subsequently recovering to 4.39% in November. Protein content remained stable during the first half of the year (~3.54%), but dropped to 3.41% in September (−3.7% from March), then increased again to 3.65% by the end of the year.

Average dry matter intake (DMI) rose from 22.8 kg in January to 24.5 kg in March (+7.5%), fluctuated within 23.6–24.9 kg in summer (with a minimum in August), and reached 25.3 kg by December (+10.8% compared to the start of the year). Feed conversion ratio (FCR) improved from 1.45 in January–February to 1.33 in December (−8.3%), reflecting enhanced feed utilisation efficiency.

The prevalence of mastitis during the first half of the year ranged from 0.87% to 1.17%, remaining at 1.2–1.3% in summer and autumn, but rising to 1.47% and 1.61% in November–December (85% above the minimum). Lameness remained between 3.15% and 3.73% throughout the year, except for a sharp increase in October to 4.57% (+39% compared to the previous month), followed by a decrease to 3.65% in December.

As the patterns observed in 2023 may reflect both stable herd characteristics and year-to-year variation, a detailed comparison of productivity dynamics, feed intake, and clinical indicators was performed for 2023 and 2024 (Table 1) to verify the robustness and reproducibility of effects identified using our algorithm.

Observations of indicator dynamics in 2024 confirm the overall consistency of the main trends identified in the previous year, in particular the gradual increase in milk yield and DMI (Table 1). At the same time, a reduction in DMI was observed during the summer, accompanied by a decrease in milk protein content, while the fat level remained stable. These findings suggest that the protein component may be more sensitive to heat stress, especially under conditions of reduced feed intake.

Monthly analysis (Table 2) revealed a distinct pattern of changes: the greatest re-ductions in milk protein concentration were recorded in July (−0.25%), June (−0.16%), and May (−0.13%), coinciding with periods of elevated THI and reduced DMI. In particular, July showed the sharpest increase in THI (average: +4.1 compared to 2023), along with the most pronounced drop in DMI (−1.38 kg) and protein content, confirming the combined negative effect of HS and feed nutritional deficit.

Unlike protein, milk fat content remained relatively stable, with minor seasonal fluctuations, including increases in August (+0.22%) and December (+0.13%), which may indicate a slightly lower sensitivity to THI effects. During the summer months, under elevated THI values, indicators of welfare deteriorated. In August and July, the frequency of lameness increased by +0.96% and +0.90%, respectively, while the incidence of mastitis slightly decreased (−0.06% in August and −0.03% in July), showing the highest increases in December (+1.04%) and April (+1.02%). Thus, the annual dynamics in 2024 are generally consistent with the findings from the previous year, con-firming the key role of heat load and feed intake as major determinants of productivity and welfare variation.

In 2024, average milk yields during the winter and spring periods were significantly higher than in 2023 (winter: 34.8 ± 0.79 kg vs. 33.3 ± 0.51 kg, *p* = 0.0412; spring: 35.6 ± 0.32 kg vs. 34.3 ± 0.51 kg, *p* = 0.0036), while no significant changes were observed in summer or autumn. Milk protein content declined in summer (down to 3.31 ± 0.05% in 2024 vs. 3.49 ± 0.08% in 2023, *p* = 0.0218), and DMI was significantly higher in winter and spring (winter: 26.0 ± 0.60 kg vs. 23.9 ± 1.27 kg, *p* = 0.0387; spring: 25.5 ± 0.67 kg vs. 24.5 ± 0.51 kg, *p* = 0.0499), but remained unchanged in the second half of the year.

Clinical indicators exhibited a more complex dynamic: mastitis frequency in 2024 increased markedly during the spring (1.77 ± 0.42% vs. 1.10 ± 0.22%, *p* = 0.0612) and winter (1.84 ± 0.68% vs. 1.26 ± 0.36%), while lameness was more frequent in winter (4.36 ± 0.44% vs. 3.51 ± 0.53%, *p* = 0.0398) and summer (4.00 ± 0.50% vs. 3.18 ± 0.38%, *p* = 0.0257). The lowest lameness rates were observed in spring, in contrast to the summer and autumn periods. Increases in mastitis and lameness were often detected after the end of heat stress periods, indicating a cumulative and delayed effect of heat load (“lag effect”). Accordingly, even after normalisation of weather conditions, cow productivity and health status may remain compromised as a result of the prolonged impact of summer heat.

To comprehensively interpret the annual and seasonal dynamics described above, the subsequent analytical tier focused on the systematic integration of production and meteorological data, including the temperature–humidity index (THI). This allowed for detailed characterisation of weather patterns, the frequency and duration of heat stress episodes, and the distribution of days by THI categories, laying the foundation for subsequent analyses.

### 3.2. Analysis of Heat Stress Episodes and Thermal Environment Dynamics

These results confirm the pivotal role of heat load, particularly maximum THI, in shaping the variation in milk quality indicators and dry matter intake. To further elucidate the nature of this effect, we analysed the frequency and duration of periods when housing conditions exceeded the thermal comfort threshold (Table 3).

In 2024, compared to 2023, there was an increase in the number of days with mild and moderate heat stress (notably for maximum THI: +21 days). The number of days with severe stress remained unchanged (nine days for maximum THI).

Analysis of prolonged periods of heat load (Table 4) showed that in 2024, heat waves were not only more frequent but also considerably longer. The longest heat wave lasted 32 days (27 June–28 July), whereas in 2023 the maximum duration was only 10 days. Moreover, days with moderate heat stress were recorded in September (11–15 September), indicating that elevated heat load persisted beyond the traditional summer months.

These findings suggest that the duration and recurrence of heat waves—rather than isolated peaks in THI—may have been the critical factors underpinning the restructuring of associations between productivity and welfare indicators, as later discussed in the correlation analysis.

Given the biological importance of nocturnal recovery, we further assessed two relative indices of heat load: the night/day THI ratio and the min/max THI ratio. The night/day THI ratio reflects the extent of temperature and humidity decrease during the night (21:00–06:00) compared to daytime values, thus indicating the effectiveness of nocturnal cooling. The min/max THI ratio, which characterises the amplitude of daily heat load fluctuations, is technically easier to compute and applicable even in the absence of hourly data. Values approaching 1 for both indices indicate minimal night-time recovery or dampened daily fluctuation.

Monthly analysis showed that the night/day THI ratio was broadly similar between years, except for a marked increase in September 2024 (+5.6% compared to 2023), pointing to reduced night cooling and a prolonged period of elevated heat load at the onset of autumn. The min/max THI ratio was higher in summer (June: +2.7%, July: +0.5%), suggesting a reduction in daily thermal amplitude at a time when cows would have benefited from greater night-time relief (Table 5).

### 3.3. Identification of Associations Between Productivity, Welfare, and THI

Correlation analysis was conducted to clarify the relationships between productivity traits, welfare indicators, and the temperature–humidity index (THI), with the aim of justifying the selection of predictors for subsequent modelling. The analysis focused on both average and maximum THI values as well as the main herd-level indicators: milk yield, milk composition, feed intake, mastitis, and lameness (Table 6).

In 2023, strong negative correlations were observed between THI and milk fat content (r = −0.828 for average THI, r = −0.825 for maximum THI, both *p* = 0.0000). Milk protein content was moderately negatively associated with THI (r = −0.452 and −0.468, *p* = 0.0000). Feed conversion ratio (FCR) showed a positive correlation with THI (r = 0.456 and 0.444, *p* = 0.0000), reflecting reduced efficiency of dry matter utilisation under heat stress. Notably, average milk yield per cow showed a strong positive correlation with THI (r = 0.669 and 0.673, *p* = 0.0000) in 2023, likely reflecting the simultaneous increase in both productivity and heat load during the year as well as compensatory management interventions such as mechanical ventilation. However, dry matter intake (DMI) did not exhibit a significant correlation with THI in 2023 (r = −0.091 and −0.087, *p* = 0.0819 and 0.0977).

Among welfare indicators, weak but statistically significant negative correlations were found between THI and the frequency of mastitis (r = −0.142 and −0.155, *p* = 0.0063 and 0.0029) and lameness (r = −0.157 and −0.149, *p* = 0.0026 and 0.0042), possibly reflecting delayed health consequences of climatic stress. Additional correlations were detected between DMI and FCR (r = −0.682), and between FCR and milk fat (r = −0.539), highlighting the importance of feed efficiency in determining milk quality. Weak positive associations were also observed between milk fat content and both mastitis (r = 0.121) and lameness (r = 0.317).

By 2024, the impact of heat load on productivity and welfare intensified. Milk protein content and DMI demonstrated even stronger negative correlations with both average and maximum THI (protein: r = −0.895 and −0.886; DMI: r = −0.724 and −0.697, all *p* = 0.0000). The correlation between THI and milk fat content remained strongly negative but was slightly less pronounced (r = −0.781 and −0.790). Importantly, the correlation between milk yield and THI shifted from positive in 2023 to weakly negative in 2024 (r = −0.075 and −0.044, *p* = 0.1512 and 0.3951), indicating a reduced tolerance of cows to heat load and possible exhaustion of adaptive capacity. The association between THI and mastitis became notably stronger (r = −0.462 and −0.466, *p* = 0.0000), while the relationship with lameness remained weak and inconsistent.

Correlations among productivity, feeding, and welfare indicators in 2024 further substantiated the rationale for predictor selection. The positive association between milk protein and DMI became substantially stronger (r = 0.735, Δ = +0.601 compared to 2023), and the negative correlation between DMI and lameness also intensified (r = −0.036, Δ = −0.332). Both mastitis and lameness showed stronger negative associations with milk yield in 2024, supporting the hypothesis of delayed and cumulative effects of heat stress on herd health and performance.

Analysis of the derived indices showed that in 2024, both the night/day THI ratio and min/max THI ratio became significantly stronger predictors of productivity and welfare outcomes. For the night/day THI ratio, the negative association with milk yield increased (r = −0.173 in 2024 vs. −0.135 in 2023, *p* < 0.05), while a significant positive correlation with lameness emerged (r = 0.299 in 2024 vs. 0.078 in 2023, *p* < 0.05). Similarly, the min/max THI ratio developed a notable negative correlation with milk yield (r = −0.209 in 2024 vs. 0.084 in 2023, *p* < 0.05) and with DMI (r = −0.207 in 2024 vs. −0.008 in 2023, *p* < 0.05), as well as a markedly stronger positive association with lameness (r = 0.324 in 2024 vs. 0.007 in 2023, *p* < 0.05). These findings highlight the importance of both sustained high night-time temperatures and reduced diurnal temperature amplitude in driving cumulative heat stress effects on productivity and welfare. In 2023, positive correlations between THI and milk yield may appear counterintuitive but can be explained by a simultaneous seasonal increase in productivity and temperature under effective ventilation. In 2024, this relationship reversed, becoming negative for milk yield, while associations with milk components, which respond more rapidly to heat load, strengthened further. These shifts indicate the depletion of adaptive capacity and the cumulative impact of prolonged heatwaves, which altered the direction and magnitude of correlations across years.

### 3.4. Selection of Predictors for Further Modelling

The selection of variables for the general linear model (GLM) was based on a combination of correlation analysis and one-way ANOVA results, with priority given to indicators that demonstrated stable associations across both study years and clear biological relevance in the context of heat stress. This approach enabled the identification of predictors that captured both direct and delayed effects of THI on productivity and welfare at the herd level.

Among environmental variables, maximum THI (THI max) emerged as the most informative indicator. It consistently showed strong negative correlations with milk fat and protein (r = −0.79 to −0.89, *p* < 0.001) and a steady decline in DMI under increased heat load. This effect was confirmed by ANOVA results. In 2023, THI max explained up to 19% of milk yield variation (*p* < 0.001) and over 55% of fat variation (*p* < 0.001. In 2024, its influence on protein and DMI increased to approximately 67% and 60%, respectively (both *p* < 0.001). These findings suggest that peak temperatures rather than average values predominantly determine the intensity of heat stress. In contrast, average THI accounted for less than 10% of the variance in most indicators (*p* > 0.05) and showed unstable correlations, while the THI min/max ratio remained weak and statistically insignificant even during heatwave peaks.

The model also included the night-to-day THI ratio, which reflects the intensity of nocturnal heat load. Although its correlations with productivity were moderate (r ≈ −0.17 for milk yield in 2024, *p* < 0.05), this index was independently associated with welfare impairments. Its explanatory power reached approximately 7% for lameness (*p* < 0.01) and 6% for milk protein (*p* < 0.05). This highlights the role of insufficient night-time cooling as a driver of chronic heat stress, which is not captured by daily average THI values.

Among physiological variables, dry matter intake (DMI) was identified as a key predictor. Its correlation with protein content reached r = 0.73 (*p* < 0.001), which aligned with the proportion of explained variance reaching 12% in 2023 and over 10% in 2024 (both *p* < 0.001). DMI was also negatively correlated with lameness (r ≈ −0.33, *p* < 0.05), suggesting an indirect effect of heat stress through feed intake reduction and resulting energy deficits.

The clinical indicators mastitis and lameness remained important for characterising long-term stress effects. In 2023, THI max explained only 0.3% of mastitis variation (*p* > 0.05), but this increased to approximately 16% in 2024 (*p* < 0.001). Similarly, the effect of night-time heat load on lameness nearly doubled, reaching about 7% (*p* < 0.01), which supports their role as delayed markers of cumulative heat exposure.

Feed conversion ratio (FCR) was excluded from the model due to its strong dependence on DMI (r = −0.68, *p* < 0.001), decreased year-to-year stability (with explained variance dropping from approximately 29% to 10%), and inconsistent correlations with productivity indicators. FCR loses diagnostic value during periods of heat stress, when both milk yield and feed intake are simultaneously affected.

The final GLM model therefore included THI max, the night-to-day THI ratio, DMI, mastitis and lameness. These predictors combined high statistical significance (*p* < 0.05 to *p* < 0.001), biological plausibility and interannual stability. This configuration enhanced the model’s sensitivity to core mechanisms of heat stress, including peak temperatures, nocturnal heat load, appetite suppression and cumulative welfare deterioration.

Inclusion of calendar season as a categorical predictor was also justified both statistically and biologically. According to two-way ANOVA results, season accounted for substantial proportions of variance in key indicators in 2024. These included up to 22% for milk yield (*p* < 0.001), 31% for fat content (*p* < 0.001), 19% for mastitis (*p* < 0.01) and over 60% for lameness (*p* < 0.001). Correlation analysis further confirmed seasonal shifts in THI relationships with productivity. During spring and summer, negative associations with yield, protein and DMI strengthened or inverted, while autumn and winter were characterised by weak or positive associations. This confirms that seasonality is not only a temporal context but also an independent source of variation reflecting the combined effects of photoperiod, heatwave duration, feeding changes and management practices. At the same time, THI as a continuous variable provides an objective measure of actual heat load, which does not always align with calendar seasons. Therefore, the inclusion of both season and THI was expected to enhance the model’s capacity to capture underlying bioecological dynamics while avoiding oversimplification.

It is important to emphasise that neither one-way ANOVA nor pairwise correlations can fully account for the complex, interdependent effects of environmental, productivity and clinical factors at the herd level. These analyses serve to define the potential scope of predictors suitable for multivariate modelling. Final variable selection for GLM was based on their statistical significance, biological relevance, absence of multicollinearity and the specific conditions of 2024, characterised by prolonged heatwaves and cumulative lagged effects.

### 3.5. Modelling the Impact of THI on Dairy Cow Productivity and Welfare Using GLM

Identifying the relationship between heat stress parameters (THI), productivity dynamics, feed intake, and the incidence of clinical disorders within the herd is essential for understanding the complex adaptive processes in dairy farming. At the final stage of our unified five-tier analytical framework, following basic correlation and factor analysis, a general linear model (GLM) was applied. This approach enables a comprehensive assessment of both direct and delayed (lagged) effects of THI and animal welfare indicators.

GLM modelling was conducted separately for the main herd indicators including milk yield, milk composition, dry matter intake, and the incidence of mastitis and lameness using data from two years that differed substantially in weather conditions (2023 and 2024). Particular attention was paid to lag effects, which allow the detection of not only immediate but also delayed consequences of heat stress. A summary of GLM results is presented in Table 7, while detailed estimates for individual indicators with included lag predictors are provided in the Appendix A.

In 2023, maximum THI values were the main factor driving a rapid decline in milk yield (η^2^ = 53.1%, *p* < 0.0001), which became evident within the first week following peak heat (Table A1). In 2024, the immediate effect of THI max weakened (η^2^ = 2.3%, *p* = 0.004), while the role of accumulated heat load increased. The reduction in milk yield became significant not immediately but with a delay, appearing 60 days after prolonged heat waves (β = −0.0282, *p* < 0.0001; η^2^ = 16%, Table A2). This indicates a shift in the dominant stress type, where the prolonged exposure to elevated temperatures rather than individual peak days became the key determinant.

For milk fat, in 2023, THI max was the primary negative factor (η^2^ = 68.6%, *p* < 0.0001), with the decline observed immediately after heat events. An additional, although smaller, contribution came from the nocturnal component (THI night/day: η^2^ = 1.3%, *p* = 0.03). In 2024, the negative effect of THI max persisted but was notably reduced (η^2^ = 37.1%, *p* < 0.0001), while the prolonged, cumulative impact of heat stress (after 30–60 days) gained greater importance (Table A4).

In the case of milk protein, a moderate effect of THI max was observed in 2023 (η^2^ = 20.3%, *p* < 0.0001) without a significant role of the night-time component (*p* = 0.61). In contrast, in 2024, the negative effect intensified (η^2^ = 49.45%, *p* < 0.0001), and a significant contribution of nocturnal heat load appeared (THI night/day: η^2^ = 1.99%, *p* = 0.0072). The most pronounced decrease in protein content during 2024 occurred 30–60 days after prolonged heat waves (Table A6).

In 2023, the association between THI max and dry matter intake (DMI) was statistically insignificant (*p* = 0.98; η^2^ ≈ 0%). In 2024, it became a key determinant (η^2^ = 47.4%, *p* < 0.0001). Even a short period of heat was accompanied by a sharp reduction in feed intake, and this effect persisted after the end of the heat wave, particularly after 7–30 days (β = −0.0611 to −0.0496, *p* < 0.0001, η^2^ up to 48.1%, Table A8). Sustained elevation of night-time THI further intensified this response.

In 2023, an increase in THI produced only minimal or short-term effects (β = −0.0025, *p* = 0.013; η^2^ = 1.7% within 7 days). In 2024, mastitis incidence increased mainly 60 days after prolonged elevation of THI (β = +0.0175, *p* < 0.0001; η^2^ = 18.2%, Table A10). Therefore, the predominant mechanism shifted from acute to prolonged, delayed effects of thermal load.

In 2023, a short-term rise in THI was associated with a slight decrease in lameness (β = −0.0077, *p* = 0.0006, η^2^ = 3.3% after 7 days), but after 60 days the direction of the effect reversed, leading to an increase in lameness frequency (β = +0.0053, *p* = 0.038, η^2^ = 1.4%). In 2024, the strongest rise in lameness occurred 60 days after long-lasting heat waves (β = +0.0439, *p* < 0.0001, η^2^ = 42.2%, Table A12), showing a clear dependence on nocturnal THI levels.

These results confirm that the impact of heat stress on herd productivity and health has both direct and cumulative delayed components. In 2024, the principal factors leading to decreased productivity, altered milk composition, and increased mastitis and lameness incidence were prolonged heat waves and elevated night-time THI. The most pronounced adverse effects appeared with a delay of 30–60 days after sustained thermal stress. This highlights the need for dynamic welfare monitoring that accounts for the duration and recurrence of heat waves and explains why traditional models demonstrated lower accuracy and reproducibility under the formation of pronounced lagged effects in 2024.

A comparative analysis of overall model characteristics for 2023 and 2024 (Table 8 and Table 9) showed that the emergence of delayed (lagged) effects of heat stress in 2024 substantially complicated prediction and reduced model reproducibility. This was particularly evident for milk yield, dry matter intake, and clinical indicators, where R^2^ and predictive accuracy on independent datasets decreased markedly. These findings indicate that the cumulative and delayed nature of heat stress disrupts standard short-term associations and gives rise to more complex, nonlinear relationships that are difficult to capture within classical regression models. Consequently, under prolonged heat waves, predictive accuracy decreases sharply, and models require adaptation to account for lagged-effect dynamics.

The shift in the relative importance of individual predictors in 2024 clearly reflects the dominance of prolonged lagged heat stress effects. For instance, while in 2023 the direct impact of THI max explained the major variation in milk yield (η^2^ = 53.1%), its contribution dropped sharply in 2024 (to 2.3%), while the importance of delayed effects and clinical disturbances increased. For mastitis and lameness as predictors of milk components, the proportion of explained variance in 2024 increased up to tenfold compared to 2023, indicating an accumulation of stress effects over time.

Comparative evaluation of model performance in 2023 and 2024 revealed a significant decline in the predictive capacity of classical GLMs under the presence of lagged effects. For example, milk yield was modelled reasonably well in 2023 (R^2^ = 0.61) but dropped to 0.44 in 2024. The most pronounced drop in accuracy was seen in cross-year testing scenarios: the DMI model trained on 2023 data explained only 7% of the variability when applied to 2024 (R^2^ = 0.07), with MAE nearly doubling. The largest discrepancies were observed in clinical traits (mastitis and lameness), where even in 2024 the models explained no more than 22% of the variance, while cross-year transferability of these models reduced R^2^ nearly to zero.

*Modelling results with the inclusion of a categorical season variable*

To distinguish the independent effects of heat stress (THI and derived indices) from broader seasonal variations in physiology, feeding, and herd management, seasonality was introduced as a categorical predictor (with four levels) in all final GLMs. This approach allowed for a clearer separation of the THI contribution and for testing whether the seasonal factor acts as an independent predictor of productive and clinical indicators.

Adding seasonality did not deteriorate core model performance metrics (R^2^, MAE, RMSE); in many cases, it improved the explained variance in both training and independent subsets (Table A13, Table A14 and Table A15). For instance, milk yield R^2^ increased from 0.608 to 0.626 in 2023 and from 0.444 to 0.515 in 2024. Similar trends were found for dry matter intake, feed conversion, and clinical indicators, confirming the additional predictive value of including the seasonal factor.

It is important to emphasize that incorporating seasonality helped prevent potential conflation of THI effects with general seasonal shifts (e.g., daylength changes, feed type transitions, or shifts in reproductive status), and enhanced the transparency and accuracy of biological interpretation. Moreover, seasonal effect estimates for each trait are provided in the Appendix A (e.g., Table A13), and comprehensive model characteristics with the seasonal factor are detailed in Table A14 and Table A15.

Overall, these findings suggest that accounting for seasonality is both a statistically and biologically justified strategy for studying the impact of heat stress and its lagged effects in dairy cattle systems.

## 4. Discussion

The obtained results confirm that THI is a key quantitative indicator of heat load in dairy cows, explaining from 10.0% to 66.7% of the variation in productivity and welfare outcomes at the herd level. The strongest effects were observed for milk protein and fat content, with η^2^ reaching 66.7% and 55.4%, respectively. For milk yield, dry matter intake, and health-related indicators such as mastitis and lameness, the influence of THI also remained substantial, although in some cases it was partly exceeded by calendar-based seasonality. These findings suggest that both factors are complementary: THI provides a more dynamic and precise reflection of actual microclimatic conditions, whereas seasonality captures additional contextual variation not directly linked to thermal load [16,17,18].

In the final GLMs, only maximum THI and the THI night/day ratio were retained as robust thermal predictors, while average THI and the THI min/max ratio were excluded due to their lower explanatory power and inconsistent associations across years. This result is consistent with earlier studies indicating that average THI represents general thermal background, whereas maximum THI better reflects acute heat stress peaks that directly affect productivity and welfare [19,20,21]. In our analysis, maximum THI showed the strongest predictive capacity for milk yield, fat, and protein, confirming its utility as an indicator of peak thermal load. Although this index primarily captures short-term physiological stress, the inclusion of lagged predictors in the GLM framework allowed for the identification of delayed effects that appeared several weeks after the initial exposure. Similar delayed relationships between THI max and productive traits have been reported previously, highlighting the cumulative nature of thermal load and its prolonged metabolic impact [22,23,24]. Therefore, the combined use of maximum THI and lag-based temporal analysis provides a comprehensive means of capturing both immediate and cumulative consequences of heat stress episodes.

The THI night/day ratio, which reflects the degree of nocturnal cooling, was retained because of its high physiological relevance for thermoregulatory recovery and immune resilience. A limited night-time temperature drop hinders metabolic restitution, maintains elevated body temperature overnight, and exacerbates chronic stress responses in dairy cows [22,23,24]. In our models, a higher THI night/day ratio was significantly associated with lower milk protein content and increased mastitis incidence, particularly during prolonged summer heatwaves in 2024, consistent with previous reports linking insufficient nocturnal recovery to impaired udder health and productivity losses [25,26]. These findings confirm that nocturnal cooling acts as an independent risk factor and justify the inclusion of this ratio in integrated heat-stress assessments aimed at improving both welfare and productivity management in dairy herds.

Seasonal analysis confirmed that calendar-based seasonality remains a significant background factor influencing productivity and welfare indicators. However, its effects did not always align with periods of actual thermal load. In our GLMs, the inclusion of seasonality improved model fit for some outcomes, particularly mastitis and lameness (up to 16.1%), but its predictive capacity for milk yield and composition was consistently lower than that of THI parameters. This supports earlier observations that calendar seasons do not fully capture the variability of heat exposure in naturally ventilated systems [15,20,25]. For example, productivity losses were often more pronounced in July–August than in September–October, despite similar THI values, indicating a cumulative thermal burden that extends beyond strict seasonal boundaries [27]. This phenomenon, also known as autumn productivity depression, may reflect a delayed endocrine and metabolic imbalance following prolonged heatwaves. These findings highlight the need to treat season as a contextual modifier rather than a primary predictor when assessing HS effects under conditions of climatic instability.

Reduced dry matter intake (DMI) is a central pathway mediating productivity losses under heat stress. When THI exceeds 72, DMI typically declines by 8–12%, directly contributing to reduced milk yield [24,28]. However, this decline is not solely due to feed avoidance behaviour but also reflects deeper metabolic adaptations. Elevated cortisol concentrations during HS suppress gastrointestinal motility, lower dry matter digestibility by 8–15%, and shift energy allocation away from lactation [24,29]. This induces a negative energy balance, mobilisation of fat reserves, and activation of ketogenesis, often accompanied by changes in the fat-to-protein ratio (FPR) in milk, which is a known indicator of metabolic stress [30]. Hence, productivity decline during heat exposure results from both reduced feed intake and impaired nutrient utilisation, driven by stress-related endocrine and metabolic responses. Our findings support this interpretation, as maximum THI explained nearly 60% of the variation in DMI at the herd level, confirming the sensitivity of feeding behaviour and digestive efficiency to acute thermal load.

Heat stress directly compromises animal health by increasing the risk of clinical mastitis and lameness. The incidence of mastitis may rise by 30–50% during hot periods due to systemic immunosuppression triggered by activation of the hypothalamic–pituitary–adrenal axis and elevated cortisol levels. These changes reduce the activity of neutrophils and T lymphocytes and suppress the expression of genes involved in antibacterial defence [31,32,33]. Heat stress also impairs gastrointestinal barrier function and promotes systemic inflammation, increasing susceptibility to secondary infections [34]. Lameness is similarly linked to heat-induced behavioural alterations. During periods of high THI, cows tend to stand longer, lie down less frequently, and reduce rumination time. This leads to overloading of the hooves, mechanical trauma, and inflammation [26,35,36]. As a result, the prevalence of lameness may increase by up to 60% in summer. These findings support the notion that heat stress affects animal health through a combination of immune, physiological, and behavioural pathways. In our study, THI was a statistically significant predictor of clinical outcomes, explaining 16.1% of the variation in mastitis cases and 10.0% in lameness incidence.

The proposed algorithm, which integrates correlation analysis and factorial assessment, enabled not only the identification of key predictors but also the quantification of their contribution to variation in productivity and welfare. This approach facilitates cross-herd and cross-year comparisons and allows the adaptation of recommendations to different housing and management conditions. THI proved to be the most informative external indicator of heat stress, explaining up to 49.5% of the variance in milk protein content, 68.6% in milk fat, and 47.4% in dry matter intake. Compared to calendar-based seasonality, THI showed greater sensitivity to short-term peaks in thermal load and enabled more precise detection of physiological responses within the herd [37,38,39,40].

At the same time, the impact of THI is modulated by internal herd dynamics and cumulative stress effects. In particular, clinical indicators such as mastitis and lameness accounted for an increasing proportion of variation in milk traits under prolonged heat exposure, highlighting the need for integrated models that incorporate both environmental and health-related parameters. Such models enhance the explanatory power of THI-based assessment and improve the applicability of decision-making strategies in herd management.

However, several methodological limitations should be acknowledged.

A limitation of this study is the exclusive use of the classical Temperature–Humidity Index (THI), which considers only air temperature and relative humidity. Although THI remains the most widely used indicator due to its simplicity and the broad availability of the required data, it does not account for other critical environ-mental factors such as solar radiation or air movement. Recent studies have demonstrated that more comprehensive thermal indices, such as the Black Globe Humidity Index (BGHI) and the Comprehensive Climate Index (CCI), which incorporate radiation and wind speed, show stronger correlations with physiological indicators of heat stress in dairy cows, especially under naturally ventilated conditions [41]. However, the use of these indices was not feasible in our study due to the absence of black globe temperature and wind speed data in the available meteorological records. Future re-search should prioritize the collection of on-farm microclimatic data and the application of more complex indices to enhance the biological relevance of heat stress assessment at the herd level.

Another limitation of this study is the reliance on meteorological data from a regional weather station rather than direct on-farm measurements. Previous research has shown that microclimatic conditions inside naturally ventilated barns can differ substantially from those measured at nearby weather stations, due to the influence of building design, ventilation, animal density, and management practices [42]. The absence of on-farm microclimatic measurements could therefore have reduced the precision of associations observed between environmental factors and animal responses. Future studies should prioritize direct on-farm data collection to improve the accuracy and biological relevance of environmental monitoring.

A further limitation of this study is the absence of physiological parameters such as respiration rate, rectal temperature, or skin temperature. These direct indicators of heat stress are important for the biological validation of environmental models and for understanding the actual welfare status of dairy cows [43]. Without such measure-ments, the association between environmental indices and animal well-being remains less substantiated. Future research should include relevant physiological data to strengthen the reliability and practical relevance of herd-level heat stress assessment.

Finally, the single-farm design and moderate effect sizes observed for certain clinical outcomes (e.g., mastitis, lameness) limit the generalisability of the findings. Future multicentre studies are needed to confirm the external validity of THI-based models under varying management and climatic conditions.

Practical implications and future directions

The findings of this study support a multifactorial framework for monitoring heat stress in dairy herds, with a particular focus on clinically relevant and delayed effects. Rather than relying solely on average THI, we recommend prioritising maximum daily THI as the most sensitive predictor of acute thermal load and productivity decline. Its predictive power for dry matter intake and milk composition confirms its value as a leading environmental indicator in heat stress assessment.

In addition, the THI night/day ratio may serve as a supplementary tool for evaluating the adequacy of nocturnal recovery and identifying potential cumulative effects. Although its explanatory power was lower than that of maximum THI, it may provide valuable insights into prolonged exposure patterns, particularly during extended heatwaves or transitional seasons.

The integration of THI parameters with physiological and welfare-related traits, such as DMI, mastitis, and lameness, enhances the sensitivity of predictive models and allows earlier identification of vulnerable subgroups. Our results indicate that heat stress not only impairs productivity but also compromises animal health through immunosuppression, metabolic imbalance, and altered behaviour.

Future monitoring systems should incorporate lagged THI effects and clinical indicators into generalised linear or machine learning models to support early warning and adaptive decision-making. Special consideration should be given to delayed consequences emerging after visible thermal stress subsides, especially during the late summer and early autumn periods. This approach offers a more robust and biologically relevant basis for precision management under variable climatic and housing conditions.

## 5. Conclusions

This study confirmed the effectiveness of a multi-level algorithm for quantifying the impact of heat stress on dairy cow productivity and welfare using herd-level data. Among meteorological variables, maximum daily THI showed the strongest association with key traits related to milk production and feeding behaviour. Notably, performance decline under thermal load was linked not only to reduced feed intake but also to welfare impairments such as mastitis and lameness, which emerged as significant components of the stress response. Their inclusion in multifactorial models strengthened the explanatory power of THI-based assessment and highlighted the need for clinically relevant endpoints in monitoring systems, including the evaluation of delayed effects. Future research should prioritise the development of predictive tools that integrate environmental, physiological, and behavioural parameters for early detection of heat-related risks, enabling real-time, welfare-oriented management in naturally ventilated dairy systems.

## Figures and Tables

**Table 1:** Comparison of annual average values of milk performance, DMI, welfare indicators, and THI in the dairy herd between 2023 and 2024.

Indicator	2023	2024	*p*-Value
Milk yield, kg	34.1 ± 0.77	34.9 ± 1.27	0.0000
Fat, %	4.14 ± 0.185	4.16 ± 0.156	0.7167
Protein, %	3.53 ± 0.088	3.46 ± 0.109	0.0000
FCR, kg DMI/kg milk	1.41 ± 0.053	1.38 ± 0.139	0.0000
DMI, kg	24.3 ± 0.882	25.1 ± 1.15	0.0000
Mastitis, %	1.21 ± 0.283	1.53 ± 0.055	0.0000
Lameness, %	3.54 ± 0.604	3.87 ± 0.922	0.0000
THI (average)	51.1 ± 13.62	52.0 ± 14.32	0.2183
THI (maximum)	56.6 ± 14.53	57.3 ± 15.37	0.3214

Note. FCR = feed conversion ratio; DMI = dry matter intake; THI = temperature–humidity index. FCR values are expressed as kilograms of DMI per kilogram of milk yield.

**Table 2:** Monthly variation in herd-level performance and welfare indicators in 2024 compared to 2023.

Month	Milk Yield, kg	Fat, %	Protein, %	DMI, kg	FCR	Mastitis, %	Lameness, %	THI avg	THI max
January	+2.12	+0.02	−0.01	+3.31	−0.11	+0.67	+1.00	−4.0	−3.7
February	+2.46	−0.03	−0.01	+2.54	−0.04	+0.01	+0.56	+5.1	+4.9
March	+1.86	−0.12	−0.01	+1.59	−0.01	+0.61	−0.69	−0.1	+0.1
April	+0.82	−0.06	−0.06	+0.83	−0.02	+1.02	−1.31	+4.2	+6.3
May	+1.43	+0.01	−0.13	+0.79	+0.01	+0.39	−0.80	+0.7	+1.1
June	+0.87	+0.03	−0.16	−0.06	+0.03	+0.15	+0.62	+3.5	+3.1
July	−0.81	+0.03	−0.25	−1.38	+0.06	−0.03	+0.90	+4.1	+4.0
August	+0.02	+0.22	−0.12	+0.97	−0.05	−0.06	+0.96	−2.2	−1.6
September	−0.63	+0.15	−0.03	+0.09	−0.07	−0.19	+1.36	+2.4	+0.6
October	+0.86	−0.10	+0.00	+0.31	−0.03	−0.07	−0.08	−0.3	−0.7
November	+0.71	−0.03	−0.06	+0.78	−0.06	+0.39	+0.56	−1.5	−2.6
December	+0.17	+0.13	+0.04	+0.33	+0.00	+1.04	+0.97	−3.2	+2.5

Note. Absolute monthly differences in herd-level indicators between 2024 and 2023. Positive values indicate an increase in 2024; negative values indicate a decrease.

**Table 3:** Distribution of days by heat stress categories based on average and maximum THI values in 2023–2024.

Heat Stress Category	THI Average 2023	THI Average 2024	THI Maximum 2023	THI Maximum 2024
Comfort conditions (<68)	328	311	254	228
Mild stress (68–71.9)	24	38	52	58
Moderate stress (72–79.9)	13	17	50	71
Severe stress (≥80)	0	0	9	9

Note. Classification was carried out according to four categories: comfort conditions (THI < 68), mild stress (THI 68–71.9), moderate stress (THI 72–79.9), and severe stress (THI ≥ 80). The count was performed separately for average daily and maximum THI values for each day of the year.

**Table 4:** Duration of heat stress waves (THI ≥ 72) in 2023–2024.

Year	Start Date	End Date	Duration (days)
2023	17 June 2023	25 June 2023	9
2023	1 July 2023	7 July 2023	7
2023	16 July 2023	20 July 2023	5
2023	29 July 2023	7 August 2023	10
2023	14 August 2023	21 August 2023	8
2023	25 August 2023	1 September 2023	8
2023	21 September 2023	26 September 2023	6
2024	1 June 2024	11 June 2024	11
2024	27 June 2024	28 July 2024	32
2024	6 August 2024	10 August 2024	5
2024	16 August 2024	5 September 2024	21
2024	11 September 2024	15 September 2024	5

Note. Only periods lasting at least 5 days with a daily maximum THI ≥ 72 were classified as heat stress waves. A gap of 1 day was not considered a break in the wave and did not divide it into separate periods.

**Table 5:** Monthly dynamics of night/day THI and min/max THI ratios in 2023–2024.

Month	THI Night/Day 2023	THI Night/Day 2024	Δ	THI Min/Max 2023	THI Min/Max 2024	Δ
January	0.921 ± 0.0985	0.905 ± 0.1316	−0.016	0.791 ± 0.0865	0.738 ± 0.1088	−0.053
February	0.885 ± 0.0767	0.907 ± 0.0704	+0.022	0.772 ± 0.0882	0.813 ± 0.0656	+0.041
March	0.852 ± 0.0867	0.852 ± 0.0792	0.000	0.743 ± 0.0745	0.743 ± 0.0781	0.000
April	0.896 ± 0.0666	0.859 ± 0.0778	−0.037	0.814 ± 0.0687	0.756 ± 0.0648	−0.058
May	0.842 ± 0.0421	0.835 ± 0.0536	−0.007	0.774 ± 0.0463	0.763 ± 0.0479	−0.011
June	0.879 ± 0.0472	0.895 ± 0.0448	+0.016	0.823 ± 0.0419	0.845 ± 0.0311	+0.022
July	0.891 ± 0.0483	0.890 ± 0.0309	−0.001	0.835 ± 0.0473	0.839 ± 0.0265	+0.004
August	0.883 ± 0.0373	0.865 ± 0.0362	−0.018	0.822 ± 0.0351	0.803 ± 0.0388	−0.019
September	0.861 ± 0.0433	0.909 ± 0.0430	+0.048	0.772 ± 0.0498	0.829 ± 0.0468	+0.057
October	0.902 ± 0.0835	0.919 ± 0.0785	+0.017	0.801 ± 0.0708	0.804 ± 0.0670	+0.003
November	0.931 ± 0.0922	0.934 ± 0.0985	+0.003	0.798 ± 0.0807	0.810 ± 0.0702	+0.012
December	0.937 ± 0.0744	0.957 ± 0.0512	+0.020	0.821 ± 0.0722	0.871 ± 0.0583	+0.050

Note. The night/day THI ratio reflects the effectiveness of night-time cooling, while the min/max THI ratio characterises the amplitude of daily fluctuations in heat load. Values close to 1 indicate the absence of night-time recovery or minimal daily variation.

**Table 6:** Pairwise correlation coefficients (r) for associations between THI and key performance and welfare indicators in 2023 and 2024.

Indicator	THI avg 2023	*p*-Value	THI max 2023	*p*-Value	THI avg 2024	*p*-Value	THI max 2024	*p*-Value
Milk yield, kg	0.669	0.0000	0.673	0.0000	−0.075	0.1512	−0.044	0.3951
Fat, %	−0.828	0.0000	−0.825	0.0000	−0.781	0.0000	−0.790	0.0000
Protein, %	−0.452	0.0000	−0.468	0.0000	−0.895	0.0000	−0.886	0.0000
DMI, kg	−0.091	0.0819	−0.087	0.0977	−0.724	0.0000	−0.697	0.0000
FCR, kg DMI/kg milk	0.456	0.0000	0.444	0.0000	0.278	0.0000	0.272	0.0000
Mastitis, %	−0.142	0.0063	−0.155	0.0029	−0.462	0.0000	−0.466	0.0000
Lameness, %	−0.157	0.0026	−0.149	0.0042	−0.117	0.0248	−0.165	0.0015

**Table 7:** Parameter estimates of the general linear model (GLM) for key herd indicators in 2023 and 2024.

Predictor	2023			2024		
	Estimate (95% CI)	*p*-Value	Partial η^2^ (%)	Estimate (95% CI)	*p*-Value	Partial η^2^ (%)
Milk yield, kg						
Intercept	23.1749 (21.5626, 24.7873)	<0.0001	—	25.0136 (22.2222, 27.8051)	<0.0001	—
THI, max	0.0371 (0.0335, 0.0408)	<0.0001	53.1	0.0109 (0.0035, 0.0183)	0.0040	2.3
THI, night/day	0.2781 (−0.4224, 0.9786)	0.4355	0.2	−0.3149 (−1.2523, 0.6225)	0.5090	0.1
DMI, kg	0.3794 (0.3141, 0.4447)	<0.0001	26.7	0.4654 (0.3804, 0.5504)	<0.0001	24.3
Mastitis, %	−0.1979 (−0.3968, 0.0009)	0.0511	1.1	−0.3725 (−0.5230, −0.2221)	<0.0001	6.2
Lameness, %	−0.1143 (−0.2046, −0.0240)	0.0132	1.7	−0.4120 (−0.4999, −0.3240)	<0.0001	19.1
Fat, %						
Intercept	4.2684 (3.9474, 4.5895)	<0.0001	—	4.7592 (4.3997, 5.1186)	<0.0001	—
THI, max	−0.0103 (−0.0110, −0.0096)	<0.0001	68.6	−0.0071 (−0.0080, −0.0061)	<0.0001	37.05
THI, night/day	−0.1539 (−0.2934, −0.0144)	0.0307	1.3	−0.1268 (−0.2475, −0.0061)	0.0396	1.17
DMI, kg	0.0171 (0.0041, 0.0301)	0.0103	1.8	−0.0114 (−0.0223, −0.0004)	0.0414	1.15
Mastitis, %	−0.0092 (−0.0488, 0.0304)	0.6484	0.06	0.0941 (0.0747, 0.1134)	<0.0001	20.21
Lameness, %	0.0538 (0.0358, 0.0718)	<0.0001	8.8	0.0166 (0.0052, 0.0279)	0.0043	2.25
Protein, %						
Intercept	3.5321 (3.2733, 3.7909)	<0.0001	—	3.2259 (3.0371, 3.4148)	<0.0001	—
THI, max	−0.0028 (−0.0034, −0.0022)	<0.0001	20.3	−0.0048 (−0.0053, −0.0043)	<0.0001	49.45
THI, night/day	−0.0294 (−0.1418, 0.0831)	0.6077	0.07	−0.0872 (−0.1506, −0.0238)	0.0072	1.99
DMI, kg	0.0073 (−0.0032, 0.0178)	0.1699	0.52	0.0214 (0.0157, 0.0272)	<0.0001	12.99
Mastitis, %	0.0204 (−0.0115, 0.0524)	0.2086	0.44	0.0296 (0.0194, 0.0398)	<0.0001	8.31
Lameness, %	−0.0060 (−0.0205, 0.0085)	0.4143	0.19	−0.0003 (−0.0062, 0.0057)	0.9320	0.002
DMI, kg						
Intercept	21.5789 (20.3351, 22.8228)	<0.0001	—	30.54 (29.29, 31.79)	<0.0001	—
THI, max	0.00009 (−0.0057, 0.0058)	0.976	0.02	−0.060 (−0.067, −0.054)	<0.0001	47.4
THI, night/day	−0.5349 (−1.6454, 0.5755)	0.344	0.25	−0.76 (−1.89, 0.38)	0.192	0.47
Mastitis, %	1.2412 (0.9529, 1.5295)	<0.0001	16.6	−0.25 (−0.43, −0.07)	0.0069	2.0
Lameness, %	0.4825 (0.3482, 0.6168)	<0.0001	12.2	−0.23 (−0.33, −0.12)	<0.0001	4.8
FCR, kg milk/kg DMI						
Intercept	1.3579 (1.2895, 1.4262)	<0.0001	—	1.1908 (1.1262, 1.2554)	<0.0001	—
THI, max	0.0015 (0.0012, 0.0018)	<0.0001	19.1	0.0027 (0.0024, 0.0031)	<0.0001	41.2
THI, night/day	0.1418 (0.0808, 0.2028)	<0.0001	5.5	0.0958 (0.0368, 0.1547)	0.0015	2.75
Mastitis, %	−0.0554 (−0.0712, −0.0396)	<0.0001	11.6	−0.0059 (−0.0153, 0.0034)	0.214	0.43
Lameness, %	−0.0270 (−0.0344, −0.0196)	<0.0001	12.6	−0.0089 (−0.0143, −0.0035)	0.0013	2.82
Mastitis, %						
Intercept	0.9794 (0.5822, 1.3766)	<0.0001	—	2.8655 (2.1911, 3.5400)	<0.0001	—
THI, max	−0.00253 (−0.00467, −0.00040)	0.0147	1.63	−0.0174 (−0.0208, −0.0140)	<0.0001	21.8
THI, night/day	0.4149 (0.0178, 0.8121)	0.0406	1.15	−0.3717 (−1.0363, 0.2929)	0.2721	0.33
Lameness, %						
Intercept	3.5356 (2.6830, 4.3883)	<0.0001	—	1.2227 (0.0525, 2.3930)	0.0406	—
THI, max	−0.0058 (−0.0101, −0.0014)	0.0097	1.84	−0.0058 (−0.0117, 0.0001)	0.0558	1.0
THI, night/day	0.3710 (−0.0593, 0.8013)	0.393	0.20	3.3507 (2.1976, 4.5037)	<0.0001	8.3

Note: The table presents estimates with 95% confidence intervals, *p*-values, and partial eta-squared (η^2^) values for each predictor. Negative values indicate inverse associations. Predictors include maximum THI, the ratio of night to day THI, dry matter intake (DMI), mastitis incidence, and lameness incidence. Detailed results for lagged predictors are available in the Appendix A.

**Table 8:** Summary performance metrics of regression models for main productive and clinical traits in 2023 and 2024.

Indicator	Year	Multiple R	R^2^	Adjusted R^2^	F-Stat	df	Model *p*-Value
Milk yield, kg	2023	0.779	0.608	0.602	111.27	5.359	<0.0001
	2024	0.666	0.444	0.436	57.36	5.360	<0.0001
Fat, %	2023	0.853	0.728	0.725	192.57	5.359	<0.0001
	2024	0.8424	0.7096	0.7056	175.93	5.360	<0.0001
Protein, %	2023	0.482	0.233	0.222	21.79	5.359	<0.0001
	2024	0.914	0.835	0.832	363.42	5.360	<0.0001
DMI, kg	2023	0.493	0.243	0.235	28.91	4.360	<0.0001
	2024	0.722	0.522	0.516	98.43	4.361	<0.0001
FCR, kg milk/kg DMI	2023	0.6145	0.3775	0.3706	54.59	4.360	<0.0001
	2024	0.7281	0.5301	0.5249	101.81	4.361	<0.0001
Mastitis, %	2023	0.188	0.0353	0.0300	6.62	2.362	0.0015
	2024	0.471	0.2219	0.2176	51.76	2.363	<0.0001
Lameness, %	2023	0.156	0.0243	0.0190	4.51	2.362	0.0117
	2024	0.333	0.1108	0.1059	22.62	2.363	<0.0001

**Table 9:** Accuracy of model predictions in within-year and cross-year testing (R^2^, MAE, RMSE values).

Model (Training)	Test Set	R^2^	MAE	RMSE
Milk yield, kg				
2023	2023	0.6078	0.3801	0.4835
2023	2024	0.1774	0.8373	0.9711
2024	2024	0.4434	0.5060	0.6554
2024	2023	0.3617	0.7447	0.8996
Fat, %				
2023	2023	0.6983	0.0802	0.1015
2023	2024	0.5988	0.0924	0.1129
2024	2024	0.7096	0.0666	0.0844
2024	2023	0.6592	0.0915	0.1158
Protein, %				
2023	2023	0.2328	0.0619	0.0776
2023	2024	0.8223	0.0848	0.1020
2024	2024	0.8346	0.0350	0.0444
2024	2023	0.2276	0.1036	0.1273
DMI, kg				
2023	2023	0.2431	0.5950	0.7685
2023	2024	0.0670	0.9697	1.2201
2024	2024	0.5217	0.6007	0.7991
2024	2023	0.0004	1.1518	1.5956
FCR, kg milk/kg DMI				
2023	2023	0.3775	0.0305	0.0422
2023	2024	0.4015	0.0381	0.0487
2024	2024	0.5301	0.0313	0.0414
2024	2023	0.2585	0.0340	0.0501
Mastitis, %				
2023	2023	0.0353	0.2230	0.2777
2023	2024	0.1311	0.4507	0.6296
2024	2024	0.2219	0.3751	0.4889
2024	2023	0.0204	0.3961	0.4901
Lameness, %				
2023	2023	0.0243	0.4720	0.5960
2023	2024	0.0614	0.8150	0.9479
2024	2024	0.1108	0.6640	0.8483
2024	2023	0.0129	0.5817	0.7203

## Data Availability

The data presented in this study are available on request from the corresponding author. The data are not publicly available due to confidentiality agreements with the commercial farm involved in the study.

## Abbreviations

The following abbreviations are used in this manuscript:

THI	Temperature–Humidity Index
HS	Heat Stress
DMI	Dry Matter Intake
FCR	Feed Conversion Ratio
ANOVA	Analysis of Variance
GLM	General Linear Model
CI	Confidence Interval
η^2^ₓ	Partial Eta-Squared
R^2^	Coefficient of Determination
MAE	Mean Absolute Error
RMSE	Root Mean Square Error

## Appendix A. Appendix Tables

The following supplementary tables are provided in the Appendix A and labelled as Table A1, Table A2, Table A3, Table A4, Table A5, Table A6, Table A7, Table A8, Table A9, Table A10, Table A11, Table A12, Table A13, Table A14 and Table A15, in accordance with the journal’s format. These tables contain detailed outputs of GLMs with lagged predictors, seasonal effects, and extended diagnostics that support the main findings of the manuscript.

**Table A1:** GLM estimates for milk yield (2023) including lagged effects of THI at 7-, 30-, and 60-day intervals.

Predictor	Lag 7			Lag 30			Lag 60		
	Estimate (95% CI)	*p*-Value	Partial η^2^ (%)	Estimate (95% CI)	*p*-Value	Partial η^2^ (%)	Estimate (95% CI)	*p*-Value	Partial η^2^ (%)
Intercept	22.98 (20.88, 25.09)	<0.001	56.7	29.18 (26.71, 31.64)	<0.001	62.2	33.21 (30.53, 35.89)	<0.001	66.5
THI, max	0.032 (0.028, 0.036)	<0.001	38.7	0.019 (0.014, 0.024)	<0.001	15.9	0.002 (−0.003, 0.008)	0.448	0.19
THI, night/day	−0.06 (−0.88, 0.76)	0.891	0.01	−0.92 (−1.82, −0.02)	0.045	1.2	−0.44 (−1.48, 0.60)	0.410	0.23
DMI, kg	0.42 (0.33, 0.51)	<0.001	20.6	0.27 (0.17, 0.37)	<0.001	8.1	0.13 (0.02, 0.25)	0.020	1.8
Mastitis, %	−0.28 (−0.51, −0.05)	0.016	1.6	−0.71 (−0.96, −0.46)	<0.001	8.4	−0.79 (−1.07, −0.51)	<0.001	9.4
Lameness, %	−0.14 (−0.25, −0.04)	0.008	2.0	−0.28 (−0.39, −0.16)	<0.001	6.1	−0.27 (−0.40, −0.14)	<0.001	5.2

**Table A2:** GLM estimates for milk yield (2024) including lagged effects of THI at 7-, 30-, and 60-day intervals.

Predictor	Lag 7			Lag 30			Lag 60		
	Estimate (95% CI)	*p*-Value	Partial η^2^ (%)	Estimate (95% CI)	*p*-Value	Partial η^2^ (%)	Estimate (95% CI)	*p*-Value	Partial η^2^ (%)
Intercept	26.82 (23.95, 29.69)	<0.001	48.95	31.04 (28.62, 33.46)	<0.001	65.96	32.06 (30.14, 33.97)	<0.001	78.33
THI, max	0.0026 (−0.0051, 0.0102)	0.5082	0.12	−0.0118 (−0.0306, 0.0070)	0.1329	3.08	−0.0282 (−0.0356, −0.0209)	<0.001	16.02
THI, night/day	0.486 (−0.484, 1.456)	0.3253	0.27	−0.929 (−1.905, 0.004)	0.0619	1.05	−1.122 (−2.138, −0.107)	0.0304	1.55
DMI, kg	0.401 (0.314, 0.488)	<0.001	18.87	0.318 (0.232, 0.405)	<0.001	16.16	0.294 (0.222, 0.366)	<0.001	17.65
Mastitis, %	−0.472 (−0.626, −0.318)	<0.001	9.33	−0.413 (−0.471, −0.355)	<0.001	14.89	−0.606 (−0.749, −0.464)	<0.001	18.92
Lameness, %	−0.480 (−0.566, −0.393)	<0.001	25.11	−0.414 (−0.537, −0.359)	<0.001	24.04	−0.242 (−0.362, −0.150)	<0.001	7.05

**Table A3:** GLM estimates for milk fat (%) in 2023 including lagged effects of THI at 7-, 30-, and 60-day intervals.

Predictor	Lag 7			Lag 30			Lag 60		
	Estimate (95% CI)	*p*-Value	Partial η^2^ (%)	Estimate (95% CI)	*p*-Value	Partial η^2^ (%)	Estimate (95% CI)	*p*-Value	Partial η^2^ (%)
Intercept	3.85 (3.46; 4.24)	<0.001	51.5	2.66 (2.17; 3.16)	<0.001	25.6	1.74 (1.15; 2.33)	<0.001	10.2
THI, max	−0.0097 (−0.0105; −0.0089)	<0.001	62.9	−0.0076 (−0.0085; −0.0066)	<0.001	43.1	−0.0054 (−0.0066; −0.0042)	<0.001	21.0
THI, night/day	−0.035 (−0.187; 0.118)	0.8935	0.01	−0.048 (−0.228; 0.132)	0.5998	0.08	−0.095 (−0.324; 0.133)	0.4122	0.22
DMI, kg	0.029 (0.013; 0.045)	0.0005	3.4	0.061 (0.041; 0.081)	<0.001	10.0	0.087 (0.063; 0.112)	<0.001	13.8
Mastitis, %	−0.0067 (−0.049; 0.036)	0.7576	0.04	0.101 (0.051; 0.152)	0.0001	4.5	0.182 (0.121; 0.243)	<0.001	10.2
Lameness, %	0.051 (0.031; 0.070)	<0.001	7.0	0.095 (0.071; 0.118)	<0.001	16.2	0.118 (0.089; 0.147)	<0.001	18.0

**Table A4:** GLM estimates for milk fat (%) in 2024 including lagged effects of THI at 7-, 30-, and 60-day intervals.

Predictor	Lag 7			Lag 30			Lag 60		
	Estimate (95% CI)	*p*-Value	Partial η^2^ (%)	Estimate (95% CI)	*p*-Value	Partial η^2^ (%)	Estimate (95% CI)	*p*-Value	Partial η^2^ (%)
Intercept	4.67 (4.30; 5.04)	<0.001	63.86	3.00 (2.63; 3.37)	<0.001	43.25	2.60 (2.28; 2.93)	<0.001	46.01
THI, max	−0.0069 (−0.0079; −0.0059)	<0.001	34.97	−0.0015 (−0.0027; −0.0004)	0.0063	2.24	0.0015 (0.0003; 0.0027)	0.0171	1.88
THI, night/day	−0.1094 (−0.23; 0.01)	0.085	0.84	0.073 (−0.08; 0.22)	0.343	0.27	0.1010 (−0.07; 0.27)	0.243	0.45
DMI, kg	−0.0105 (−0.0217; 0.0006)	0.064	0.97	0.0314 (0.0193; 0.0434)	<0.001	7.35	0.0390 (0.0270; 0.0511)	<0.001	11.91
Mastitis, %	0.0952 (0.0755; 0.1150)	<0.001	20.32	0.1470 (0.1235; 0.1706)	<0.001	31.42	0.1787 (0.1549; 0.2026)	<0.001	42.01
Lameness, %	0.0270 (0.0158; 0.0381)	<0.001	6.05	0.0433 (0.0304; 0.0563)	<0.001	11.65	0.0282 (0.0115; 0.0449)	0.0010	3.55

**Table A5:** GLM estimates for milk protein (%) in 2023 including lagged effects of THI at 7-, 30-, and 60-day intervals.

Predictor	Lag 7			Lag 30			Lag 60		
	Estimate (95% CI)	*p*-Value	Partial η^2^ (%)	Estimate (95% CI)	*p*-Value	Partial η^2^ (%)	Estimate (95% CI)	*p*-Value	Partial η^2^ (%)
Intercept	3.59 (3.29; 3.89)	<0.001	61.0	3.21 (2.89; 3.53)	<0.001	53.8	3.16 (2.83; 3.50)	<0.001	53.6
THI, max	−0.0026 (−0.0032; −0.0020)	<0.001	17.0	−0.0022 (−0.0028; −0.0016)	<0.001	13.0	−0.0021 (−0.0027; −0.0014)	<0.001	10.7
THI, night/day	0.043 (−0.061; 0.133)	0.4707	0.15	0.012 (−0.106; 0.130)	0.8409	0.01	−0.148 (−0.224; −0.015)	0.0251	1.7
DMI, kg	0.0014 (−0.011; 0.012)	0.8218	0.01	0.0151 (0.0022; 0.0281)	0.0218	1.6	0.0197 (0.0056; 0.0337)	0.0063	2.5
Mastitis, %	0.024 (−0.009; 0.057)	0.1501	0.59	0.0545 (0.0214; 0.0877)	0.0013	3.1	0.0776 (0.043; 0.112)	<0.001	6.0
Lameness, %	−0.0046 (−0.019; 0.010)	0.5433	0.10	−0.0014 (−0.017; 0.014)	0.8563	0.01	0.0106 (−0.0057; 0.027)	0.2009	0.54

**Table A6:** GLM estimates for milk protein (%) in 2024 including lagged effects of THI at 7-, 30-, and 60-day intervals.

Predictor	Lag 7			Lag 30			Lag 60		
	Estimate (95% CI)	*p*-Value	Partial η^2^ (%)	Estimate (95% CI)	*p*-Value	Partial η^2^ (%)	Estimate (95% CI)	*p*-Value	Partial η^2^ (%)
Intercept	2.93 (2.72; 3.15)	<0.001	67.02	2.14 (1.93; 2.34)	<0.001	55.50	1.84 (1.66; 2.03)	<0.001	55.48
THI, max	−0.0040 (−0.0046; −0.0035)	<0.001	34.97	−0.0020 (−0.0026; −0.0014)	<0.001	10.88	−0.00053 (−0.0012; 0.00019)	0.149	0.69
THI, night/day	−0.0195 (−0.09; 0.05)	0.600	0.08	0.1044 (0.02; 0.19)	0.015	1.79	0.0018 (−0.098; 0.101)	0.972	<0.01
DMI, kg	0.0274 (0.02; 0.034)	<0.001	16.17	0.0470 (0.040; 0.054)	<0.001	45.60	0.0569 (0.050; 0.064)	<0.001	45.60
Mastitis, %	0.0356 (0.024; 0.047)	<0.001	9.40	0.0548 (0.042; 0.068)	<0.001	16.99	0.0777 (0.064; 0.092)	<0.001	28.57
Lameness, %	0.0071 (0.0006; 0.014)	0.033	1.28	0.0197 (0.013; 0.027)	<0.001	6.80	0.0232 (0.013; 0.033)	<0.001	6.80

**Table A7:** GLM estimates for DMI (kg) in 2023 including lagged effects of THI at 7-, 30-, and 60-day intervals.

Predictor	Lag 7			Lag 30			Lag 60		
	Estimate (95% CI)	*p*-Value	Partial η^2^ (%)	Estimate (95% CI)	*p*-Value	Partial η^2^ (%)	Estimate (95% CI)	*p*-Value	Partial η^2^ (%)
Intercept	21.77 (20.62; 22.92)	<0.001	79.7	3.55 (3.42; 3.69)	<0.001	89.3	21.52 (20.37; 22.66)	<0.001	82.1
THI, max	−0.0021 (−0.0072; 0.0030)	0.4198	0.18	−0.0023 (−0.0029; −0.0016)	<0.001	13.5	−0.0041 (−0.0095; 0.0014)	0.1430	0.71
THI, night/day	0.075 (−0.92; 1.07)	0.8822	0.01	0.0047 (−0.114; 0.123)	0.9383	0.002	0.75 (−0.30; 1.79)	0.1600	0.65
Mastitis, %	1.00 (0.74; 1.26)	<0.001	14.2	0.066 (0.034; 0.098)	<0.001	4.8	0.81 (0.55; 1.08)	<0.001	10.9
Lameness, %	0.41 (0.29; 0.53)	<0.001	11.4	0.0046 (−0.010; 0.019)	0.5311	0.12	0.44 (0.32; 0.56)	<0.001	14.6

**Table A8:** GLM estimates for DMI (kg) in 2024 including lagged effects of THI at 7-, 30-, and 60-day intervals.

Predictor	Lag 7			Lag 30			Lag 60		
	Estimate (95% CI)	*p*-Value	Partial η^2^ (%)	Estimate (95% CI)	*p*-Value	Partial η^2^ (%)	Estimate (95% CI)	*p*-Value	Partial η^2^ (%)
Intercept	30.76 (29.53; 31.99)	<0.001	87.18	27.83 (26.40; 29.26)	<0.001	81.56	22.72 (21.15; 24.28)	<0.001	73.07
THI, max	−0.0611 (−0.0677; −0.0544)	<0.001	48.12	−0.0496 (−0.0579; −0.0412)	<0.001	29.23	−0.0174 (−0.0288; −0.0060)	0.0028	2.93
THI, night/day	−1.3447 (−2.50; −0.19)	0.0228	1.46	0.0006 (−1.35; 1.35)	0.9993	<0.01	2.4141 (0.84; 3.99)	0.0028	2.94
Mastitis, %	−0.2616 (−0.44; −0.08)	0.0052	2.19	−0.0719 (−0.28; 0.14)	0.503	0.14	0.5124 (0.30; 0.73)	<0.001	6.71
Lameness, %	−0.1253 (−0.23; −0.02)	0.0176	1.58	0.0740 (−0.04; 0.19)	0.209	0.48	0.0917 (−0.07; 0.25)	0.250	0.44

**Table A9:** GLM estimates for mastitis (%) in 2023 including lagged effects of THI at 7-, 30-, and 60-day intervals.

Predictor	Lag 7			Lag 30			Lag 60		
	Estimate (95% CI)	*p*-Value	Partial η^2^ (%)	Estimate (95% CI)	*p*-Value	Partial η^2^ (%)	Estimate (95% CI)	*p*-Value	Partial η^2^ (%)
Intercept	0.876 (0.477, 1.275)	<0.001	4.99	0.891 (0.491, 1.290)	<0.001	5.48	1.216 (0.774, 1.657)	<0.001	8.83
THI, max	−0.0025 (−0.0046, −0.0005)	0.0133	1.71	−0.0020 (−0.0041, 0.0001)	0.0672	1.01	0.0033 (0.0010, 0.0056)	0.0053	2.54
THI, night/day	0.541 (0.140, 0.942)	0.0083	1.95	0.518 (0.031, 0.246)	0.0117	1.90	−0.204 (−0.653, 0.245)	0.3729	0.26

**Table A10:** GLM estimates for mastitis (%) in 2024 including lagged effects of THI at 7-, 30-, and 60-day intervals.

Predictor	Lag 7			Lag 30			Lag 60		
	Estimate (95% CI)	*p*-Value	Partial η^2^ (%)	Estimate (95% CI)	*p*-Value	Partial η^2^ (%)	Estimate (95% CI)	*p*-Value	Partial η^2^ (%)
Intercept	2.8398 (2.1712, 3.5084)	<0.001	16.39	2.8122 (2.1389, 3.4855)	<0.001	16.86	1.7954 (1.0043, 2.5865)	<0.001	6.18
THI, max	−0.01860 (−0.02203, −0.01518)	<0.001	24.28	−0.02161 (−0.02519, −0.01803)	<0.001	29.75	−0.01753 (−0.02172, −0.01333)	<0.001	18.22
THI, night/day	−0.2597 (−0.9188, 0.3993)	0.4388	0.17	0.0009 (−0.6719, 0.6737)	0.9979	0.00	0.9344 (0.1297, 1.7391)	0.0230	1.69

**Table A11:** GLM estimates for lameness (%) in 2023 including lagged effects of THI at 7-, 30-, and 60-day intervals.

Predictor	Lag 7			Lag 30			Lag 60		
	Estimate (95% CI)	*p*-Value	Partial η^2^ (%)	Estimate (95% CI)	*p*-Value	Partial η^2^ (%)	Estimate (95% CI)	*p*-Value	Partial η^2^ (%)
Intercept	4.47 (3.60, 5.34)	<0.001	22.5	3.76 (2.88, 4.63)	<0.001	19.1	2.87 (1.90, 3.84)	<0.001	10.1
THI, max	−0.0077 (−0.0121, −0.0033)	0.0006	3.3	−0.0034 (−0.0079, 0.0012)	0.1501	0.6	0.0053 (0.0003, 0.0104)	0.0380	1.4
THI, night/day	−0.55 (−1.42, 0.33)	0.2187	0.4	0.007 (−0.109, 0.110)	0.9876	≈0	0.42 (−0.56, 1.41)	0.3970	0.2

**Table A12:** GLM estimates for lameness (%) in 2024 including lagged effects of THI at 7-, 30-, and 60-day intervals.

Predictor	Lag 7			Lag 30			Lag 60		
	Estimate (95% CI)	*p*-Value	Partial η^2^ (%)	Estimate (95% CI)	*p*-Value	Partial η^2^ (%)	Estimate (95% CI)	*p*-Value	Partial η^2^ (%)
Intercept	0.529 (−0.655, 1.712)	0.3804	0.22	0.181 (−1.045, 1.409)	0.7713	0.03	−0.385 (−1.479, 0.709)	0.4889	0.16
THI, max	−0.00052 (−0.00658, 0.00554)	0.8672	<0.01	0.0165 (0.00993, 0.02299)	0.0000	6.88	0.0439 (0.0381, 0.0497)	0.0000	42.22
THI, night/day	3.794 (2.628, 4.961)	0.0000	10.31	3.034 (1.808, 4.261)	0.0000	6.64	1.743 (0.630, 2.855)	0.0022	3.04

**Table A13:** GLM estimates for all outcome indicators (milk yield, fat, protein, DMI, FCR, mastitis, and lameness) in 2023 and 2024 including seasonal factor.

Indicator	Predictor	2023 Estimate (95% CI)	2023 *p*-Value	2023 Partial η^2^ (%)	2024 Estimate (95% CI)	2024 *p*-Value	2024 Partial η^2^ (%)
Milk yield, kg	Intercept	22.11 (20.28, 23.94)	<0.0001	—	23.12 (20.41, 25.82)	<0.000001	—
	THI, max	0.04 (0.03, 0.04)	<0.0001	28.1	0.01 (0.002, 0.019)	0.0205	1.5
	THI, night/day	0.47 (−0.24, 1.17)	0.193	0.5	0.25 (−0.65, 1.15)	0.581	0.09
	DMI, kg	0.41 (0.34, 0.48)	<0.0001	26.0	0.49 (0.40, 0.58)	<0.000001	25.5
	Mastitis, %	−0.18 (−0.40, 0.03)	0.0511	0.8	−0.47 (−0.61, −0.32)	0.00000052	10.3
	Lameness, %	−0.08 (−0.17, 0.02)	0.106	0.7	−0.17 (−0.30, −0.05)	0.0064	2.1
	Season (factor)	—	—	4.7	—	—	12.9
	Season 1	0.04 (−0.11, 0.18)	0.594	—	−0.05 (−0.23, 0.14)	0.63	—
	Season 2	0.12 (0.03, 0.21)	0.0103	—	0.44 (0.25, 0.63)	0.00001	—
	Season 3	0.03 (−0.11, 0.17)	0.675	—	0.12 (−0.07, 0.30)	0.21	—
Fat, %	Intercept	4.5932 (4.2387, 4.9477)	<0.000001	—	4.8356 (4.4711, 5.2002)	<0.000001	—
	THI, max	−0.01154 (−0.01278, −0.01030)	<0.000001	48.42	−0.0064 (−0.0075, −0.0052)	<0.000001	24.6
	THI, night/day	−0.09265 (−0.22883, 0.04353)	0.182	0.50	−0.1679 (−0.2889, −0.0470)	0.0066	2.05
	DMI, kg	0.00318 (−0.01066, 0.01701)	0.652	0.057	−0.0111 (−0.0229, 0.0007)	0.0642	0.96
	Mastitis, %	0.01849 (−0.02098, 0.05795)	0.358	0.24	0.0988 (0.0795, 0.1181)	<0.000001	22.1
	Lameness, %	0.05256 (0.03473, 0.07038)	0.000000015	8.63	−0.0077 (−0.0243, 0.0089)	0.3637	0.23
	Season (factor)	—	—	9.94	—	—	4.69
	Season 1	−0.05898 (−0.08697, −0.03099)	0.0000427	—	0.0321 (0.0074, 0.0568)	0.0111	—
	Season 2	0.04843 (0.03086, 0.06600)	0.00000011	—	−0.0487 (−0.0747, −0.0227)	0.00027	—
	Season 3	0.00540 (−0.02126, 0.03205)	0.691	—	−0.0117 (−0.0366, 0.0133)	0.3586	—
Protein, %	Intercept	3.7040 (3.4204, 3.9877)	<0.0001	—	3.4476 (3.2740, 3.6212)	<0.000001	—
	THI, max	−0.0051 (−0.0061, −0.0041)	<0.0001	22.2	−0.0046 (−0.0051, −0.0040)	<0.000001	42.44
	THI, night/day	−0.0226 (−0.1316, 0.0863)	0.6834	0.05	−0.1017 (−0.1593, −0.0441)	0.00058	3.27
	DMI, kg	0.0041 (−0.0070, 0.0151)	0.4706	0.15	0.0126 (0.0070, 0.0182)	0.000013	5.21
	Mastitis, %	0.0217 (−0.0099, 0.0533)	0.1779	0.51	0.0348 (0.0256, 0.0440)	<0.000001	13.43
	Lameness, %	0.0017 (−0.0126, 0.0159)	0.8176	0.01	−0.0023 (−0.0102, 0.0056)	0.5647	0.09
	Season (factor)	—	—	11.26	—	—	21.76
	Season 1	−0.0609 (−0.0833, −0.0385)	<0.0001	—	−0.0004 (−0.0122, 0.0114)	0.9443	—
	Season 2	0.0199 (0.0059, 0.0340)	0.0056	—	0.0037 (−0.0087, 0.0160)	0.5621	—
	Season 3	0.0546 (0.0332, 0.0759)	<0.0001	—	−0.0358 (−0.0477, −0.0240)	<0.000001	—
DMI, kg	Intercept	22.52 (21.25, 23.80)	<0.0001	—	27.9831 (26.6045, 29.3618)	<0.0001	—
	THI, max	−0.0104 (−0.0197, −0.0011)	0.0285	1.3	−0.0374 (−0.0469, −0.0279)	<0.0001	14.41
	THI, night/day	−0.14 (−1.16, 0.89)	0.7925	0.02	−0.2958 (−1.3632, 0.7715)	0.5860	0.08
	Mastitis, %	1.25 (0.99, 1.52)	<0.0001	19.3	−0.2149 (−0.3841, −0.0457)	0.0130	1.71
	Lameness, %	0.28 (0.15, 0.41)	<0.0001	4.7	−0.0225 (−0.1692, 0.1241)	0.7626	0.03
	Season (factor)	—	—	19.7	—	—	16.87
	Season 1	−0.56 (−0.77, −0.36)	<0.0001	—	0.2655 (0.0490, 0.4820)	0.0164	—
	Season 2	0.25 (0.12, 0.38)	0.00018	—	0.4456 (0.2207, 0.6705)	<0.0002	—
	Season 3	−0.14 (−0.34, 0.06)	0.158	—	−0.8023 (−1.0059, −0.5986)	<0.0001	—
FCR, kg milk/kg DMI	Intercept	1.2890 (1.2168, 1.3612)	<0.0001	—	1.2365 (1.1669, 1.3062)	<0.0001	—
	THI, max	0.0021 (0.0016, 0.0026)	<0.0001	14.8	0.0018 (0.0013, 0.0022)	<0.0001	12.72
	THI, night/day	0.1364 (0.0783, 0.1945)	<0.0001	5.6	0.1043 (0.0504, 0.1582)	<0.0001	3.89
	Mastitis, %	−0.0525 (−0.0676, −0.0374)	<0.0001	11.5	−0.0122 (−0.0208, −0.0037)	0.0051	2.17
	Lameness, %	−0.0171 (−0.0246, −0.0097)	<0.0001	5.5	−0.0056 (−0.0130, 0.0018)	0.136	0.62
	Season (factor)	—	—	14.5	—	—	20.92
	Season 1	0.0273 (0.0159, 0.0388)	<0.0001	—	−0.0121 (−0.0230, −0.0011)	0.0307	—
	Season 2	−0.0039 (−0.0113, 0.0034)	0.2930	—	0.0037 (−0.0077, 0.0150)	0.528	—
	Season 3	0.0027 (−0.0086, 0.0141)	0.6362	—	0.0369 (0.0267, 0.0472)	<0.0001	—
Mastitis, %	Intercept	1.4295 (0.9697, 1.8720)	<0.0001	—	2.0901 (1.3674, 2.8128)	<0.0001	—
	THI, max	−0.0202 (−0.0354, −0.0058)	0.000091	4.18	−0.0171 (−0.0227, −0.0116)	<0.0001	9.30
	THI, night/day	−0.0711 (−0.2376, 0.0950)	0.403	0.27	0.4776 (−0.1815, 1.1367)	0.155	0.56
	Season (factor)	—	—	5.77	—	—	14.3
	Season 1	−0.1982 (−0.4144, −0.0453)	0.00020	—	−0.0187 (−0.1487, 0.1112)	0.777	—
	Season 2	0.0939 (−0.1493, 0.2376)	0.205	—	0.2838 (0.1976, 0.3701)	<0.0001	—
	Season 3	0.1347 (0.0596, 0.2098)	0.00047	—	0.0055 (−0.1192, 0.1303)	0.930	—
Lameness, %	Intercept	3.1082 (2.2068, 4.0095)	<0.0001	—	4.0443 (3.2105, 4.8781)	<0.0001	—
	THI, max	−0.00092 (−0.0081, 0.0063)	0.8021	0.018	−0.0049 (−0.0113, 0.0015)	0.131	0.63
	THI, night/day	0.5433 (−0.2681, 1.3546)	0.1887	0.49	0.1445 (−0.6160, 0.9050)	0.709	0.04
	Season (factor)	—	—	15.20	—	—	62.1
	Season 1	−0.0643 (−0.2240, 0.0955)	0.4295	—	0.3679 (0.2179, 0.5178)	<0.0001	—
	Season 2	0.0701 (−0.0308, 0.1711)	0.1728	—	−1.2094 (−1.3090, −1.1099)	<0.0001	—
	Season 3	−0.3380 (−0.4910, −0.1851)	0.0000181	—	0.2058 (0.0618, 0.3497)	0.0052	—

**Table A14:** Model performance statistics (R, R^2^, adjusted R^2^, F-statistic, and model *p*-value) for GLMs with seasonal factors in 2023 and 2024.

Indicator	Year	Multiple R	R^2^	Adjusted R^2^	F-Stat	df	Model *p*-Value
Milk yield, kg	2023	0.791	0.626	0.618	74.60	8.356	<0.0001
	2024	0.718	0.515	0.504	47.39	8.357	<0.0001
Fat, %	2023	0.8691	0.7554	0.7499	137.43	9.355	<0.0001
	2024	0.8504	0.7232	0.7170	116.60	9.356	<0.0001
Protein, %	2023	0.565	0.320	0.308	20.86	8.356	<0.0001
	2024	0.9331	0.8706	0.8677	300.28	8.357	<0.0001
DMI, kg	2023	0.626	0.392	0.380	32.93	7.357	<0.0001
	2024	0.7761	0.6024	0.5946	77.48	7.358	<0.0001
FCR, kg milk/kg DMI	2023	0.6840	0.4679	0.4575	44.85	7.357	<0.0001
	2024	0.7927	0.6284	0.6211	86.49	7.358	<0.0001
Mastitis, %	2023	0.302	0.091	0.078	7.19	5.359	<0.0001
	2024	0.577	0.333	0.324	35.99	5.360	<0.0001
Lameness, %	2023	0.4150	0.1722	0.1607	14.94	5.359	<0.000001
	2024	0.8143	0.6631	0.6584	141.69	5.360	<0.0001

**Table A15:** Cross-year validation metrics (R^2^, MAE, RMSE) for GLMs trained on 2023 and 2024 datasets, with and without seasonal adjustment.

Outcome	Model (Training)	Test Set	R^2^	MAE	RMSE
Milk yield, kg	2023	2023	0.6158	0.3788	0.4812
	2023	2024	0.2036	0.7704	0.9079
	2024	2024	0.4512	0.4985	0.6635
	2024	2023	0.3758	0.7996	0.9337
Fat, %	2023	2023	0.7553	0.0736	0.0914
	2023	2024	0.5979	0.0900	0.1125
	2024	2024	0.6362	0.0761	0.0949
	2024	2023	0.5641	0.0998	0.1257
Protein, %	2023	2023	0.3147	0.0576	0.0734
	2023	2024	0.6538	0.0867	0.1071
	2024	2024	0.8539	0.0347	0.0432
	2024	2023	0.2070	0.1073	0.1327
DMI, kg	2023	2023	0.3454	0.5605	0.7263
	2023	2024	0.1332	0.9400	1.2080
	2024	2024	0.6012	0.5467	0.7299
	2024	2023	0.0145	1.1160	1.5154
FCR, kg milk/kg DMI	2023	2023	0.3607	0.0311	0.0433
	2023	2024	0.3448	0.0411	0.0509
	2024	2024	0.5865	0.0298	0.0400
	2024	2023	0.2666	0.0334	0.0481
Mastitis, %	2023	2023	0.0190	0.9720	1.0262
	2023	2024	0.2364	1.3184	1.4053
	2024	2024	0.2893	0.3717	0.4726
	2024	2023	0.0041	0.4610	0.5627
Lameness, %	2023	2023	0.1205	0.4385	0.5735
	2023	2024	0.0271	0.9197	1.0353
	2024	2024	0.5718	0.4923	0.6104
	2024	2023	0.0069	0.8113	0.9310
